# Hybrid Steel Fiber Design in Ultra-High-Performance Concrete Containing Coarse Aggregate Using Pore Size Distribution Within Coarse Aggregate Skeleton

**DOI:** 10.3390/ma19061248

**Published:** 2026-03-21

**Authors:** Rui Tang, Yinfei Du, Jian Zhang, Lingxiang Kong

**Affiliations:** 1 School of Civil Engineering, Central South University, Changsha 410075, China; 234811175@csu.edu.cn (R.T.); yfdu_csu@csu.edu.cn (Y.D.); 2 Central & Southern China Municipal Engineering Design and Research Institute Co., Ltd., Wuhan 430010, China; 17872788936@163.com; 3 Technology Department, Hunan Automotive Engineering Vocational University, Zhuzhou 412001, China

**Keywords:** ultra-high-performance concrete, steel fibers, CT image analysis, workability, mechanical properties

## Abstract

To address the challenge of coarse aggregates hindering steel fiber dispersion and reducing toughening efficiency in ultra-high-performance concrete containing coarse aggregate (UHPC-CA), this study proposes a hybrid fiber design method based on reverse adaptation to the aggregate structure: a paradigm where fiber proportions are inversely designed to match the quantified void size distribution within the coarse aggregate skeleton. Industrial X-ray computed tomography (X-CT) was employed to capture the internal structure of UHPC-CA. Digital image processing techniques were used to quantitatively characterize the size distribution within the coarse aggregate skeleton gap. Based on this distribution, the blending proportions of multi-scale (3–16 mm) copper-plated steel fibers were systematically determined. Three fiber configurations were compared: mono-sized 13 mm fibers (Type A), an empirical model based on aggregate size (Type B), and a quantitatively designed blend based on skeleton gap distribution (Type C). At the same fiber volume fraction, the mechanical property test results show that the C type achieves approximately 18.6% higher flexural strength and 29.1% higher splitting tensile strength compared to the A type, while showing 5.3% and 6.7% improvements over the B type, and the compressive strength also increased slightly (about 3.0%). The microanalysis further confirms that the fiber distribution in the C-type design was more uniform, and the bridging effect and crack resistance were more sufficient. The proposed gap-adaptive fiber design paradigm offers an effective approach for optimizing reinforcement distribution in composites, providing theoretical and practical value for high-performance UHPC-CA applications.

## 1. Introduction

Ultra-high-performance concrete (UHPC) has become a research focus in modern civil engineering materials due to its exceptional mechanical properties and durability [1,2,3]. To further improve its economic efficiency and regulate the comprehensive properties of the material, researchers have recently attempted to introduce coarse aggregates into UHPC, resulting in the formation of ultra-high-performance concrete with coarse aggregate (UHPC-CA) [4,5]. This method demonstrates clear advantages in reducing material costs, increasing elastic modulus, and controlling shrinkage, while also helping to reduce cement usage and enhance material sustainability [6,7].

However, the addition of coarse aggregate also changes the internal structure of the material and causes a series of new problems, the most prominent of which is the spatial competition and mutual interference between coarse aggregate and steel fiber [8,9,10]. In UHPC systems without coarse aggregate, steel fibers can be uniformly distributed within the matrix, fully utilizing their toughening effect. In contrast, in UHPC-CA, the coarse aggregates occupy a significant portion of the space and form a rigid skeleton, which substantially hinders the uniform dispersion of fibers [9,11,12,13]. During the mixing and forming stages, a rigid framework forms between coarse aggregates, which hinders steel fibers from penetrating the narrow inter-aggregate spaces. As a result, the fibers tend to cluster on the aggregate surfaces, leading to localized agglomeration [14,15]. The fiber’s function is weakened, cracks cannot be effectively suppressed, and the overall toughening effect of the composite is limited, thus restricting improvement in the macro-mechanical properties of UHPC-CA [16,17]. Current studies have attempted to improve fiber dispersion by adjusting fiber parameters or optimizing aggregate gradation. The core limitation lies in the lack of a quantitative understanding of the actual internal structure of UHPC-CA. Fiber design remains at the empirical passive incorporation stage, failing to achieve spatial adaptation with the framework gaps. Single-dimensional optimization strategies are insufficient to optimize the spatial relationship between fibers and aggregates [18,19,20]. Therefore, designing fiber systems that match the internal spatial structure of aggregates has become a key approach to enhance the performance of UHPC-CA. In this paper, a design method for steel fiber based on the gap of the skeleton is studied. The aggregate skeleton gap refers to the three-dimensional interstitial spaces between coarse aggregate particles—distinct from matrix pores—that are available for steel fiber distribution.

To solve the above problems, a fiber quantitative design method based on the internal structure of UHPC-CA is proposed in this study. First, the true internal structure of the material was obtained through computed tomography (CT), and the skeleton gaps were accurately identified and measured by combining digital image processing techniques (image binarization, watershed segmentation algorithm, and equivalent ellipse analysis) [21,22]. Based on this, the system designs the complex blending ratio of multi-scale fibers (3–16 mm) to improve the spatial compatibility between fibers and the matrix. The influence of steel fiber content and design method on the working properties, compressive strength, flexural strength and splitting tensile strength of UHPC-CA was studied and combined with X-CT and optical microscopy to analyze its mechanism of action. Shifting the paradigm from “property-driven” to “space-adaptive” reinforcement, this study proposes a novel structure-guided hybrid fiber design that uses X-CT to quantify aggregate skeleton voids and inversely design fiber proportions to overcome the spatial exclusion effect in UHPC-CA. The fiber space-adaptive design methodology developed in this study not only provides a novel approach for enhancing the performance of UHPC-CA, but also offers methodological reference for the optimization design of other steel fiber composite materials.

## 2. Research Aims and Significance

### 2.1. Aims and Scope

This study aims to develop and validate a novel hybrid steel fiber design method for UHPC-CA based on quantitative characterization of the internal aggregate skeleton structure. The specific objectives are (1) to establish a CT-based image processing protocol for identifying and quantifying the three-dimensional interstitial gaps within the coarse aggregate skeleton; (2) to inversely design multi-scale steel fiber proportions (3–16 mm) that match the quantified gap size distribution; (3) to experimentally evaluate the workability and mechanical properties (compressive, flexural, and splitting tensile strengths) of UHPC-CA with the proposed fiber design; (4) to compare the performance of the gap-adaptive design (Type C) against conventional mono-fiber (Type A) and empirical hybrid (Type B) approaches; and (5) to validate the improved fiber distribution and reinforcement mechanisms through microstructural analysis.

The scope of this study is limited to UHPC-CA with a fixed coarse aggregate content (20% by volume) and optimized gradation using the Modified Andreasen and Andersen (MAA) model. Copper-plated steel fibers with lengths ranging from 3 mm to 16 mm (diameter 0.22 mm) are investigated. Fiber volume fractions are varied from 1.0% to 3.0% to identify the optimal dosage.

### 2.2. The Novelty of This Research

The novelty of this work lies in its fundamental shift from empirical, property-driven fiber design to a structure-guided, space-adaptive paradigm. Unlike previous studies that select fiber proportions based on trial-and-error or simple coordination with aggregate size, this research introduces a quantitative inverse design methodology: the fiber blend is systematically determined by the actual void size distribution within the aggregate skeleton, as captured by X-ray computed tomography. Key innovations include (1) the use of watershed segmentation and equivalent ellipse analysis to quantitatively characterize skeleton gaps; (2) the principle of “reverse adaptation”—matching fiber length distribution to gap size frequency; (3) the demonstration that short fibers (3–8 mm) preferentially populate fine interstitial spaces while long fibers (10–16 mm) bridge larger gaps, creating a hierarchical reinforcement network; and (4) experimental validation that this space-adaptive approach achieves superior fiber distribution uniformity and mechanical performance compared to conventional design methods.

### 2.3. Practical Importance

The practical importance of this research is twofold. First, the proposed gap-adaptive design method provides engineers and material designers with a rational, reproducible framework for optimizing fiber reinforcement in UHPC-CA—a material increasingly used in bridges, high-rise buildings, and infrastructure rehabilitation. By maximizing the toughening efficiency of steel fibers, the method enables (a) achievement of higher flexural and tensile strengths at the same fiber volume fraction, reducing material costs; (b) improved workability, facilitating easier placement in complex formworks; and (c) more uniform fiber distribution, enhancing structural reliability and durability. Second, the methodology is transferable to other fiber-reinforced composites where spatial constraints between reinforcements and aggregates limit performance. The CT-based characterization and inverse design approach can be adapted for different aggregate types, gradations, and fiber materials, offering a generalizable tool for advancing high-performance construction materials.

## 3. Materials and Methods

### 3.1. Materials

Type P·II 52.5R rapid-hardening Portland cement was used, and its key technical characteristics are presented in Table 1.

The other cementitious materials used include Class F fly ash with a density of 2.3 g/cm^3^, silica fume, and S95 grade ground granulated blast furnace slag (GGBS). Their primary chemical compositions are provided in Table 2.

A high-performance polycarboxylate ether-based superplasticizer with a water-reducing efficiency greater than 35% was employed. Its content was fixed at 3.0% by mass of the total cementitious materials.

The fine aggregate was natural quartz sand with a maximum particle size of 2.36 mm. It had a high SiO_2_ content of ≥99%, an apparent density of 2660 kg/m^3^, a crushing rate of ≤0.35%, and a mud content of ≤1%.

Limestone crushed stone, sieved into different size fractions, was used as the coarse aggregate. Its apparent density and water absorption for each fraction are presented in Table 3. To achieve a continuous particle size distribution spanning from the fine aggregate (2.36 mm) up to 16 mm, the lower limit of the coarse aggregate was operationally defined as 2.36 mm in this study [23].

Straight, copper-coated steel fibers were used. The fibers were 3–16 mm in length and 0.22 mm in diameter, with a density of 7850 kg/m^3^, a tensile strength greater than 2850 MPa, and an elastic modulus of 200 GPa.

### 3.2. Specimen Preparation

The UHPC-CA was prepared according to the following procedures. First, the cementitious materials and fine aggregates were poured into a cement paste mixer and dry-mixed for 2 min. Subsequently, water and superplasticizer were added, and the mixture was blended for another 3 min. Afterwards, the coarse aggregate and steel fiber were uniformly dispersed into the mixture, followed by an additional 3 min of mixing. The freshly mixed paste was then cast into molds and covered with plastic film. After curing for 24 h, the specimens were demolded and subsequently cured in a standard curing chamber maintained at 20 °C with a relative humidity greater than 95% until they reached the designated testing ages for mechanical property evaluation.

### 3.3. Physical and Mechanical Property Tests

All tests were conducted in accordance with the Chinese National Standard “Standard for test methods of concrete physical and mechanical properties” (GB/T 50081-2019) [24]. The workability was evaluated by measuring the slump and flow spread. For the compressive strength test, 100 mm × 100 mm × 100 mm cube specimens were used and loaded at a rate of 1.2 MPa/s. The flexural strength test was performed on 100 mm × 100 mm × 400 mm prisms with a loading rate of 0.1 MPa/s. The splitting tensile strength test was measured using 100 mm × 100 mm × 100 mm cubes at a loading rate of 0.1 MPa/s. All specimens underwent standard curing for 28 days before mechanical testing.

All mechanical tests (compressive, flexural, and splitting tensile strength) were conducted on a minimum of three identical specimens for each mix proportion. The results are presented as the mean value.

### 3.4. X-Ray Computed Tomography (X-CT) Scanning and Optical Microscopy Techniques

The internal microstructure of the specimens was characterized using X-CT and optical microscopy. X-ray CT scanning was performed to non-destructively reconstruct the three-dimensional internal structure based on the attenuation of X-ray intensity through materials of varying density and thickness. Optical microscopy was utilized to observe the mesoscopic structure of the UHPC-CA. The German Phoenix v|tome|xS240 industrial X-ray CT machine was selected for this study (manufactured by Waygate Technologies, sourced from Cologne, Germany). During scanning, the voltage was set at 190 kV, the current at 110 μA, and the scan interval at 0.1 mm.

## 4. Results and Discussion

### 4.1. Design of Steel Fiber Composite Based on Skeleton Gap

#### 4.1.1. Design Results of UHPC-CA Matrix and Coarse Aggregate

To establish the material basis for the steel fiber design, this paper first determines the reference mix ratio and coarse aggregate gradation of UHPC-CA. The coarse aggregate used is limestone crushed stone, and the proportion of coarse aggregate is fixed at 20%. The Modified Andreasen and Andersen (MAA) model [25,26], improved by Dinger and Funk, was employed as the classic continuous particle packing model due to its computational simplicity and reliability. This model presents the ideal packing curve corresponding to the closest packing of concrete, as expressed in Equation (1).

(1) $$P(D)=\frac{D^{q}-D_{\min}^{q}}{D_{\max}^{q}-D_{\min}^{q}}$$

where P(D) is the fraction of total solids smaller than size D; $D_{\max}$ and $D_{\min}$ are the maximum and minimum particle sizes (mm); and q is the distribution modulus, taken as 0.25 in this study.

**Figure 1:**
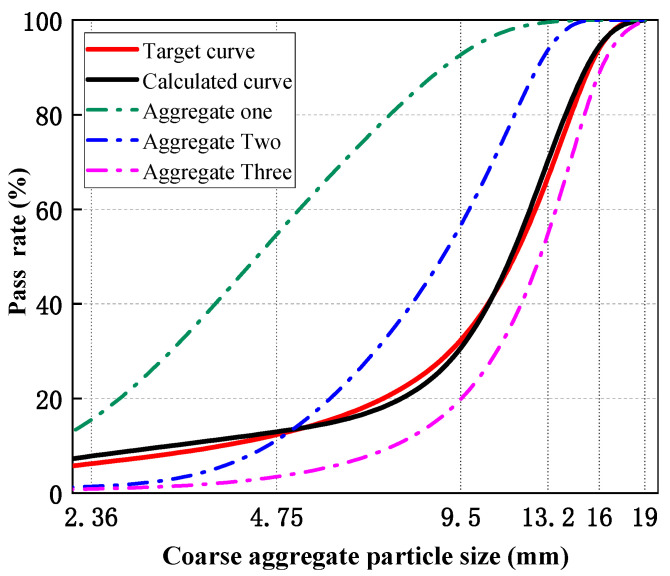
Coarse aggregate size distribution curve.

In this study, the gradation curve of the MAA model was optimally fitted based on the known particle size distribution of the aggregate. The residual sum of squares (RSS) between the target curve P(Di) and the actual distribution U(Di) was minimized using the least squares method (Equation (2)) in MATLAB software(R2024a). The blend of coarse aggregate that yielded the closest agreement with the ideal curve, as shown in Figure 1, was selected as the optimal proportion for dense packing.

(2) $$RSS=\sum_{i=1}^{n}{\left[P(D_{i})-U(D_{i})\right]}^{2}\to \min$$

The final mix proportions for UHPC containing large-sized and continuously graded coarse aggregate are detailed in Table 4.

#### 4.1.2. Design and Results of Steel Fiber Composite Reinforcement

**Figure 2:**
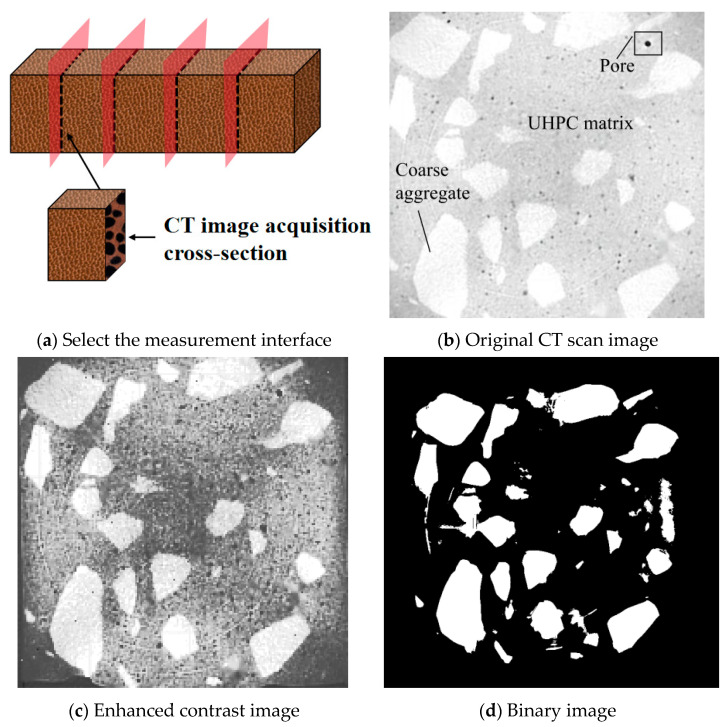
Image processing.

In order to establish the quantitative basis of the steel fiber design, this study quantitatively characterized the microstructure of a UHPC-CA matrix with a defined gradation (20% coarse aggregate volume content). The internal structure image of the specimen was obtained by industrial CT. After the original image was preprocessed by median filtering and contrast enhancement, the binary image was obtained by threshold segmentation to distinguish the coarse aggregate phase and the matrix interstitial phase. Threshold selection for phase segmentation: Threshold values were determined using Otsu’s automatic thresholding method. The grayscale histogram of the preprocessed images exhibited three distinct peaks corresponding to the following: pores: grayscale values 0–45 (darkest); the cementitious matrix: grayscale values 46–160 (intermediate); and coarse aggregates: grayscale values 161–255 (brightest). Pixels with values below 45 were classified as pores, between 46 and 160 as the matrix, and above 160 as aggregates. This tri-level thresholding enabled simultaneous segmentation of all three phases from a single image, as shown in Figure 2.

**Figure 3:**
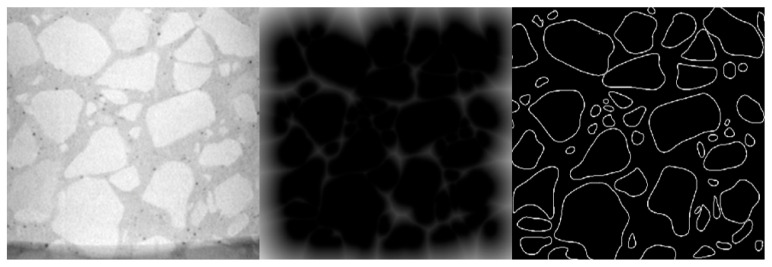
Image processing before watershed segmentation algorithm.

In the mesoscopic structure characterization of UHPC-CA, traditional image segmentation methods face difficulty in distinguishing the skeleton gap regions which are connected by the gray gradient, and it is difficult to extract the geometric parameters. Therefore, this study introduces a watershed segmentation algorithm based on the principle of morphology [27]. The algorithm maps the image gray gradient field to the terrain elevation model and simulates the convergence behavior of water flow in the gradient field according to the natural watershed boundary. The complex network of connectivity is analyzed into a series of independent units [27], as shown in Figure 3. Each independent gap unit is quantitatively characterized by the parameter “equivalent ellipse short-axis length”. This parameter can effectively measure the size of the gap and provide the geometric basis for the subsequent design of the length of the steel fiber.

**Figure 4:**
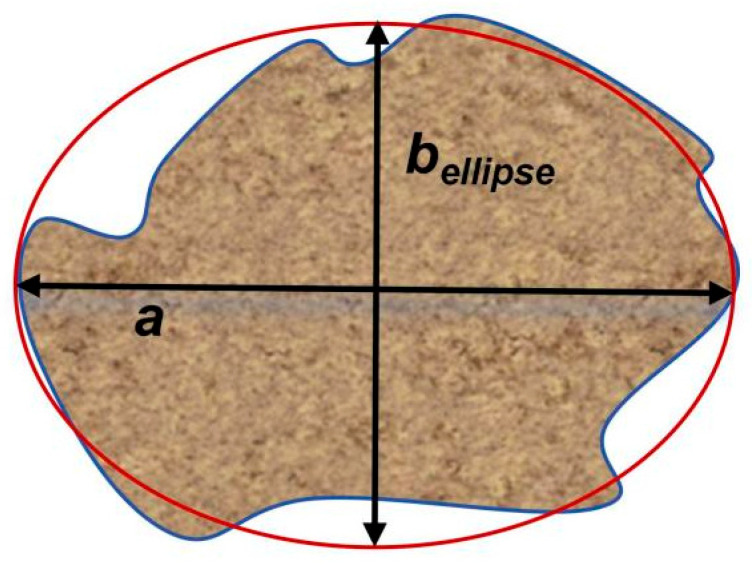
Equivalent ellipse short axis.

Due to the irregular shape and size of coarse aggregates, the equivalent ellipse short-axis length was used to characterize its dimensions, as shown in Figure 4.

(3) $$S=\pi \cdot a\cdot b_{ellipse}$$

(4) $$P=2\pi b_{ellipse}+4(a-b_{ellipse})$$

(5) $$b_{ellipse}=\frac{P-\sqrt{\pi^{2}P^{2}-32\pi S\left(\pi -2\right)}}{2\pi -4}$$

where S is the projected area of coarse aggregate; P is the projected perimeter of coarse aggregate; a is the major axis of the equivalent ellipse; and bellipse is the minor axis of the equivalent ellipse.

**Figure 5:**
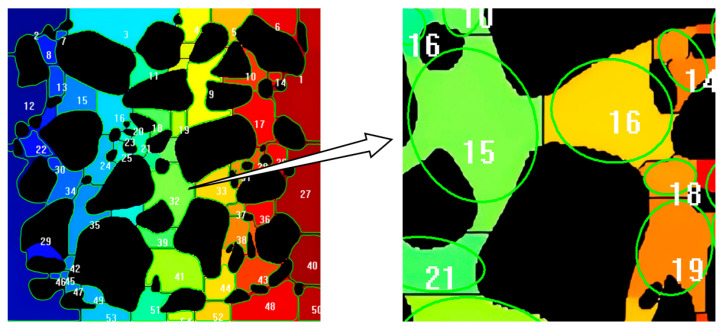
Measurement region segmentation and equivalent ellipse drawing.

We then calculated the equivalent short-axis length of all gaps and plotted the histogram of skeleton and gap dimensions (as shown in Figure 5).

Based on the distribution characteristics, the multi-scale steel fibers covering the main size range were selected, and the proportion of different lengths of fibers was determined. The results of the steel fiber mix ratio are shown in Table 5.

**Figure 6:**
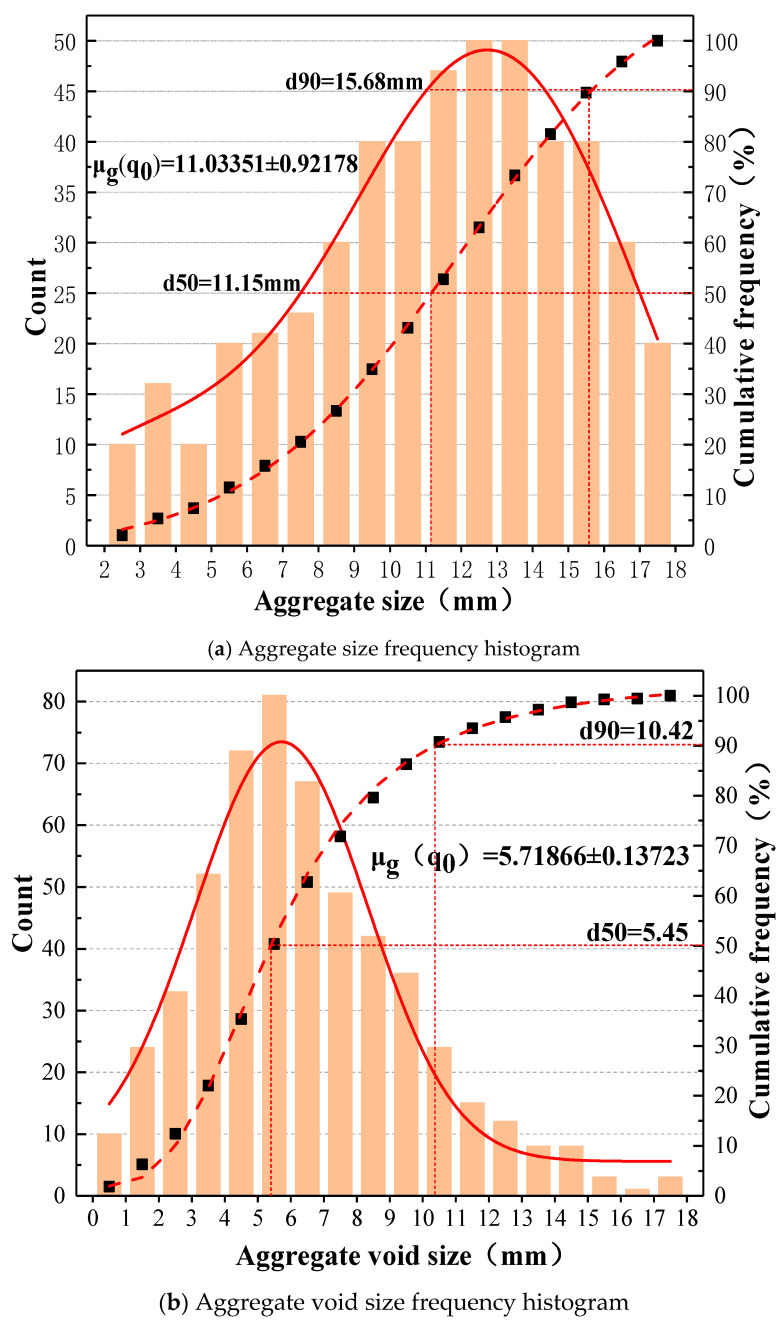
Histogram of frequency distribution of skeleton and skeleton gap.

The distribution of fiber lengths was designed to align with the frequency distribution of the equivalent ellipse short-axis lengths of the gaps. Specifically, since the gap size distribution exhibited a higher frequency in the smaller size range (approximately 3–8 mm), the fiber blend correspondingly incorporated a larger proportion of short fibers (3–8 mm) to ensure efficient filling of these narrow spaces. Conversely, as the gap size frequency decreased for larger dimensions (10–16 mm), the proportion of long fibers in the mix was reduced accordingly. This design strategy ensures that the fiber length distribution is inversely adapted to the pore structure: short fibers dominate to occupy the abundant fine gaps, while long fibers, though fewer in number, are strategically included to bridge larger interstitial regions and macro-cracks. The resulting relative proportions for the C-type mixture, as shown in Table 5, reflect this principle by assigning the highest mass fractions to the 6 mm and 3 mm fibers, which correspond to the peak of the gap size histogram. The final size statistics are shown in Figure 6.

All combinations in the UHPC-CA groups are listed in Table 6**.**

All results are reported as the mean ± standard deviation, with variability calculated from triplicate specimens. The test results of working performance and mechanical properties of all groups are shown in Table 7.

**Figure 7:**
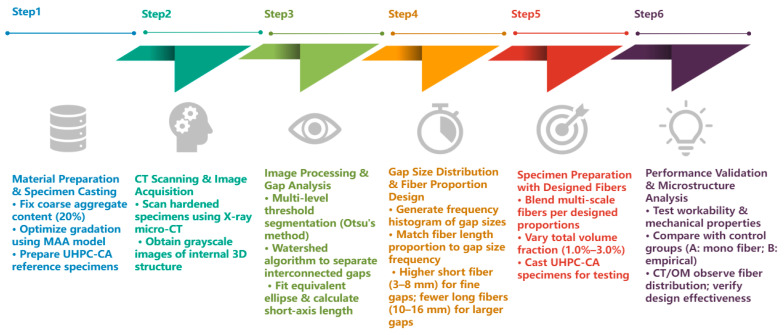
Flowchart of steel fiber design process.

The overall flowchart for steel fiber design is shown in Figure 7.

### 4.2. Effect of Steel Fiber Content on Properties of UHPC-CA

The volume ratio of steel fibers is a key factor in regulating the workability and mechanical properties of UHPC-CA. Increasing the fiber content enhances the material’s toughness, while excessive fiber content significantly reduces fluidity and weakens mechanical performance due to uneven fiber dispersion. This contradiction is particularly prominent in UHPC-CA systems, where fiber content optimization has become one of the core tasks in material design.

This study established a volume fraction gradient ranging from 1.0% to 3.0% and compared three fiber design strategies: Group A used a single fiber length (13 mm) to represent the traditional empirical approach; Group B was formulated based on the coarse aggregate particle size distribution to achieve scale coordination between fibers and aggregates; Group C was designed to match multi-scale fibers based on the quantitative characterization of skeleton gap size distribution from CT images, aiming to embed fibers more effectively into the matrix space.

#### 4.2.1. Effect of Steel Fiber Content on Working Properties of UHPC-CA

**Figure 8:**
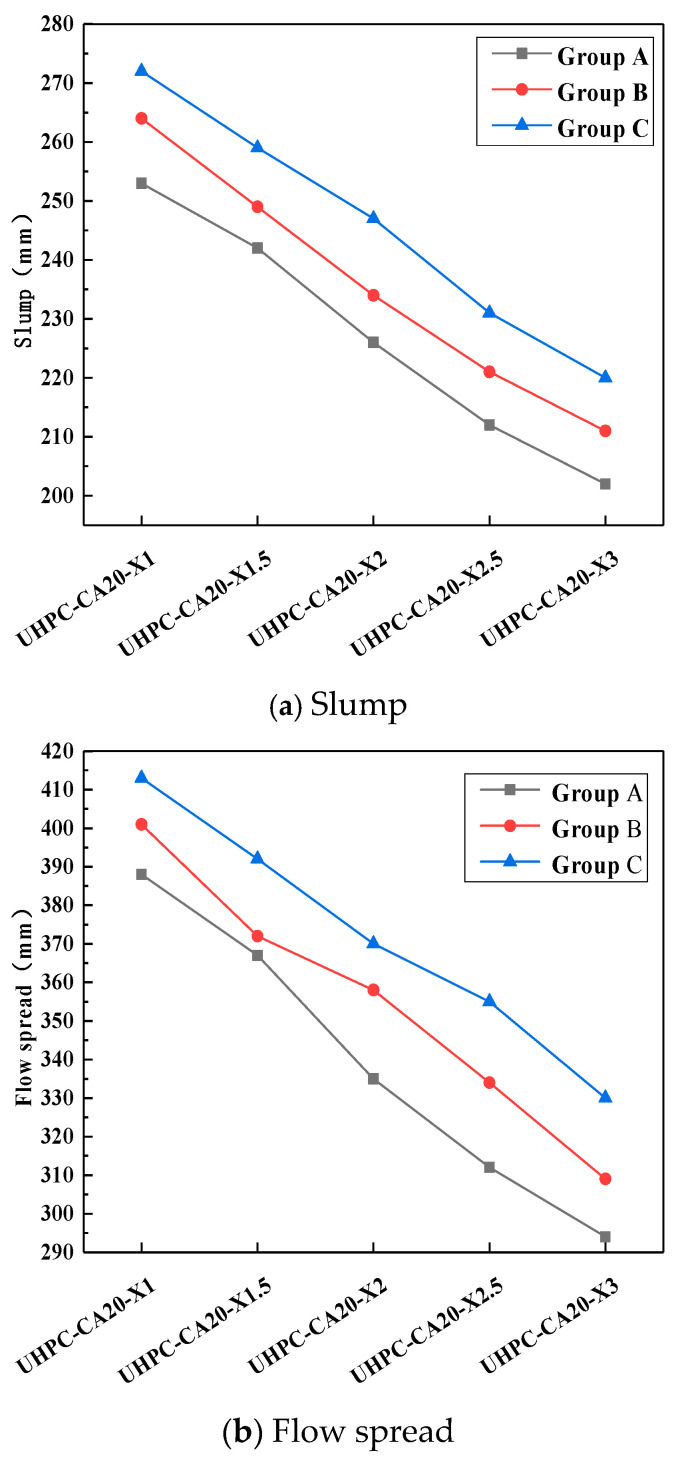
Effect of steel fiber content on workability of UHPC-CA.

As shown in Figure 8, the fluidity of UHPC-CA exhibits a linear decline with increasing steel fiber content. In Group A, the flow spread dropped sharply from 388 mm to 294 mm (a 24.0% reduction) when fiber content rose from 1% to 3%. In Group B, the flow spread decreased significantly from 408 mm to 309 mm (a 25.0% reduction) under the same fiber content range. For Group C (the gap-adaptive design), the flow spread decreased from 413 mm to 330 mm, representing a 20.1% reduction over the same increase in fiber content.

#### 4.2.2. Influence of Steel Fiber Content on Mechanical Properties of UHPC-CA

**Figure 9:**
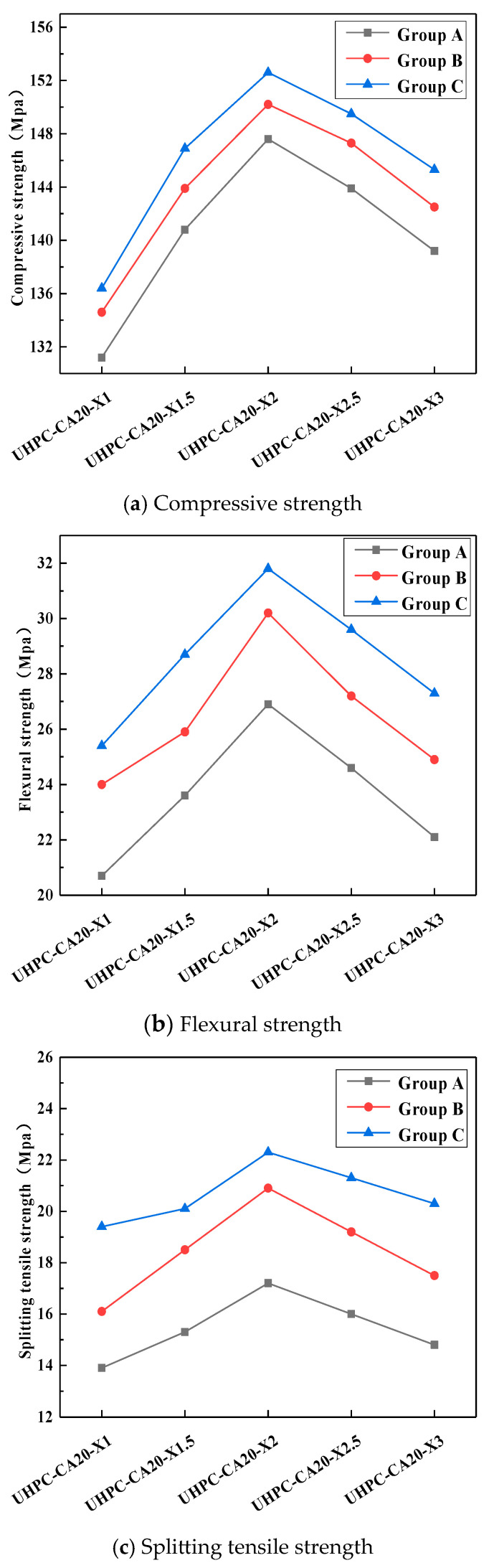
Effect of steel fiber content on mechanical properties of UHPC-CA.

The mechanical properties generally follow an upward trend followed by a decline as the steel fiber content increases, as shown in Figure 9. In terms of compressive strength, when the fiber content was gradually increased from 1% to 2%, Group A’s compressive strength rose from 131.2 MPa to 147.6 MPa, with an increase exceeding 10.0%. However, when the fiber content was further increased from 2% to 3%, the compressive strength decreased from 147.6 MPa to 139.2 MPa, showing a drop of over 5.0%. For flexural strength, when the fiber content was gradually increased from 1% to 2%, Group A’s flexural strength rose from 20.7 MPa to 26.9 MPa, with a growth of 30.0%. Conversely, when the fiber content was increased from 2% to 3%, the flexural strength decreased from 26.9 MPa to 22.1 MPa, representing a reduction of 17.8%. Regarding splitting tensile strength, when the fiber content was gradually increased from 1% to 2%, Group A’s splitting tensile strength rose from 13.9 MPa to 17.2 MPa, with a growth of 23.7%. However, when the fiber content was increased from 2% to 3%, the splitting tensile strength decreased from 17.2 MPa to 14.8 MPa, showing a drop of 14.0%.

### 4.3. Influence of Steel Fiber Design on Properties of UHPC-CA

#### 4.3.1. Influence of Steel Fiber Design on Working Properties of UHPC-CA

**Figure 10:**
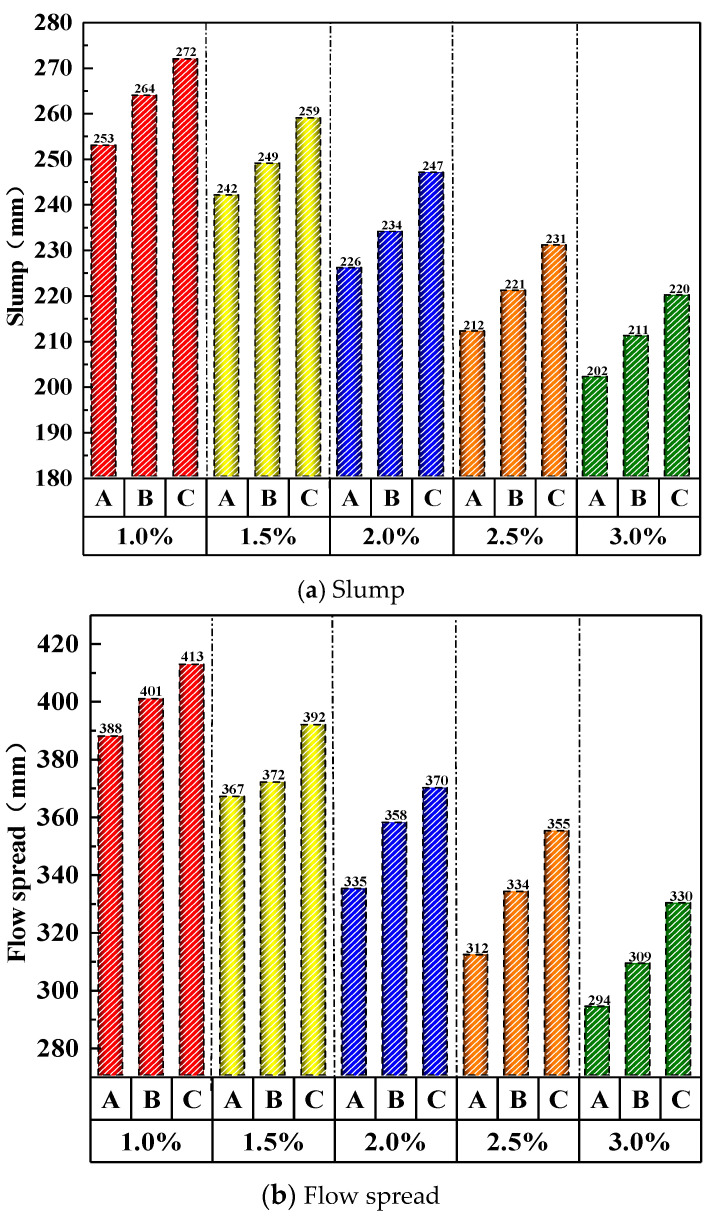
Effect of steel fiber design on workability of UHPC-CA.

As shown in Figure 10, under the same volume of admixture, the working performance ranking is as follows: Type C is the best, Type B is the second, and Type A has the worst resistance to fluidity.

#### 4.3.2. Influence of Steel Fiber Design on Mechanical Properties of UHPC-CA

**Figure 11:**
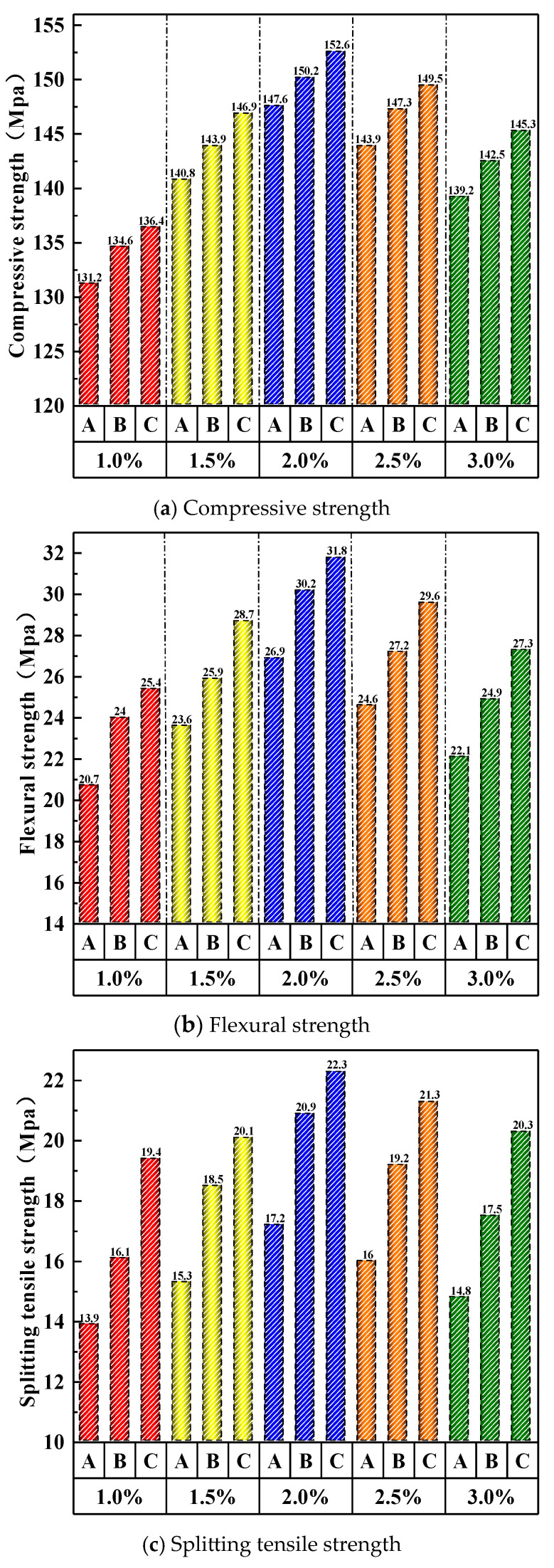
Effect of steel fiber design on mechanical properties of UHPC-CA.

The mechanical properties of UHPC-CA vary significantly depending on the steel fiber design, as shown in Figure 11. The C-type design exhibits the most significant improvements in tensile properties, including flexural and splitting tensile strength. In contrast, compressive strength is less sensitive to the fiber design, showing only minor variations, as it is primarily governed by the matrix and aggregate skeleton.

### 4.4. Analysis of the Mechanism of Damage and Enhancement

**Figure 12:**
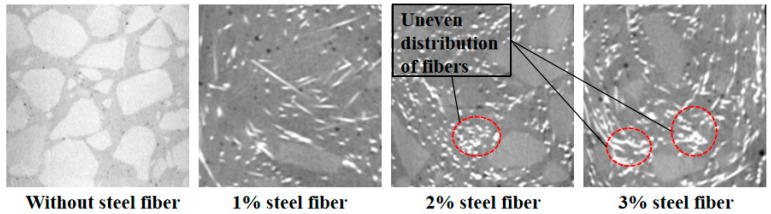
CT images of UHPC-CA with different steel fiber contents.

As shown in Figure 12, when the fiber content is high, the fibers tend to entangle and form local agglomerates, which will cause additional internal resistance and destroy the uniformity of the system. Macroscopically, the increase in fiber volume fraction compresses the flow channel of the slurry, which leads to the decrease in macroscopic flow performance. These factors can cause uneven distribution of steel fibers in concrete, thereby reducing its mechanical properties. The mechanical properties of UHPC-CA show a distinct single-peak characteristic with fiber content. At approximately 2% by volume, all mechanical properties reach their peak, where the fibers effectively bridge microcracks and inhibit their propagation. When the threshold is exceeded, the matrix defects caused by fiber agglomeration increase and the properties of the fiber are degraded. It is noteworthy that the effect of fiber content on compressive strength improvement is relatively limited, with a relatively flat trend. This is mainly because compressive performance is primarily dominated by the cement matrix and aggregate skeleton, while fibers only contribute secondary effects through lateral restraint.

**Figure 13:**
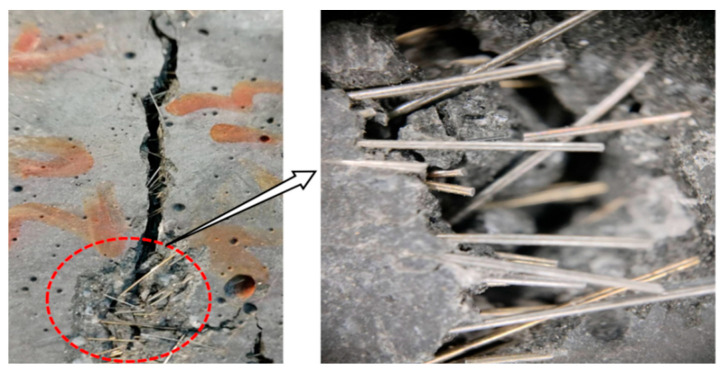
Steel fiber agglomeration.

In the C-type configuration, fibers were uniformly distributed without noticeable agglomeration, whereas the A-type configuration exhibited distinct fiber clusters and fiber-free zones. This distribution variation directly impacts mechanical properties: the uniform steel fiber arrangement forms a continuous three-dimensional reinforcement network, enabling more fibers to effectively participate in crack bridging. Conversely, fiber agglomeration leads to insufficient local reinforcement, with these fiber-free areas becoming weak points where cracks preferentially initiate and propagate, as shown in Figure 13. Thus, the enhanced mechanical performance of the C-type configuration is directly attributed to its superior fiber distribution uniformity.

In conclusion, the performance optimization of UHPC-CA requires not only precise control of fiber content but also coordinated design of its spatial distribution and matrix space.

The superior mechanical performance of the Type C design arises from three synergistic mechanisms enabled by optimized fiber distribution. First, multi-scale spatial filling: Short fibers (3–8 mm) populate narrow skeleton gaps inaccessible to longer fibers, creating a continuous reinforcement network that eliminates unreinforced zones. Second, hierarchical crack control: Short fibers, despite their lower aspect ratio and limited individual anchorage, function as micro-reinforcement in confined interstitial spaces. Their high number density enables effective dispersion and bridging of micro-cracks at the interfacial transition zone, suppressing micro-crack coalescence into macro-cracks. Concurrently, long fibers (10–16 mm) spanning larger gaps provide pull-out resistance during macro-crack propagation. Third, spatial distribution optimization: Spatial compatibility aligns fibers with principal stress trajectories, enhancing bridging efficiency. Type C specimens exhibit uniformly distributed fibers with short fibers in fine-textured regions and long fibers spanning larger cracks, whereas Type A shows fiber clustering and bare matrix zones. This hierarchical system—short fibers controlling initiation, long fibers controlling propagation and space adaptation—improves toughness and explains the 18.2% and 29.7% improvements in flexural and splitting tensile strengths, respectively, achieved by the Type C mixture.

## 5. Conclusions

Based on the above investigations, the main conclusions can be drawn:

(1) A CT-based method is proposed to quantitatively characterize skeleton gap distribution for designing multi-scale steel fibers. This structure-guided paradigm shifts fiber design from aimless experiential design to internal structural constraints, ensuring geometric compatibility in UHPC-CA where coarse aggregates create critical spatial barriers.
(2) In the UHPC-CA system, the optimal steel fiber volume fraction is 2%. Within the range of 1.0–3.0%, the material strength exhibits a unimodal trend, initially increasing and then decreasing, peaking at 2%.
(3) Group C, at a 2% volume fraction, optimizes UHPC-CA’s working performance with a 247 mm slump and 370 mm flow spread, outperforming Group A (226 mm, 335 mm) and Group B (234 mm, 358 mm). The results confirm the method’s effectiveness in improving fiber dispersion.
(4) Group C demonstrated superior mechanical properties. At a 2% volume fraction, it achieved a flexural strength of 31.8 MPa—an 18.2% increase compared to Group A and 5.3% higher than that of Group B. Its splitting tensile strength reached 22.3 MPa, rising 29.7% above that of Group A and 6.7% over that of Group B. The compressive strength was 152.6 MPa, exceeding both Groups A and B.
(5) Fiber spatial adaptation enhances comprehensive performance in UHPC-CA. Short fibers (3–8 mm) fill fine gaps, while long fibers (10–16 mm) bridge macroscopic cracks.
(6) Practical implications: The proposed gap-adaptive design method offers a rational framework for optimizing fiber reinforcement in UHPC-CA. The methodology is particularly relevant for engineering applications where UHPC-CA is subjected to tensile and flexural loads, where optimized fiber distribution directly translates to enhanced structural performance and durability.
(7) Limitations: Limitations include the following: (1) CT equipment requirement limits routine use; (2) the method assumes static aggregate structure, ignoring mixing-induced changes; (3) optimal fiber proportions are material-specific and extrapolation requires recalibration.

## 6. Future Work

To move from qualitative correlation to quantitative causation, future work should employ advanced characterization techniques. These could include nanoindentation to map the mechanical properties of the interfacial transition zone, automated image analysis to quantify fiber orientation and dispersion, and micro-CT image analysis to calculate pore structure parameters and crack density. Such quantitative data would provide definitive validation for the micromechanical models proposed herein.

## Figures and Tables

**Table 1:** Technical characteristics of cement.

Specific Surface Area (m^2^/kg)	Setting Time (min)		Compressive Strength (MPa)		Flexural Strength (MPa)	
	Initial Condensation	Final Condensation	7 d	28 d	7 d	28 d
350	116	196	39.6	60.8	6.3	9.5

**Table 2:** Chemical compositions of fly ash, silica fume, and mineral powder.

Cementitious Materials	Percentage (%)			
	SiO_2_	CaO	MgO	SO_3_
Fly ash	41.2	5.5	0.95	0.82
Silica fume	94.3	0.01	0.95	0.07
GGBS	34.2	34	6.21	1.62

**Table 3:** Density and water absorption of mineral materials.

Indicator	Nominal Particle Size (mm)			
	2.36~4.75	4.75~9.5	9.5~13.2	13.2~16
Apparent density (g/cm^3^)	2.690	2.717	2.720	2.722
Water absorption (%)	/	/	0.73	0.52

**Table 4:** UHPC-CA mix ratio.

The Proportion of Each Raw Material (kg/m^3^)							
Cement	Silica Fume	Fly Ash	Fine Aggregate	Coarse Aggregate	Steel Fiber	Superplasticizer	Water
843.5	234.3	93.7	1171.5	542.4	157.0	35.15	206.2

**Table 5:** Mass of steel fibers per cubic meter (kg/m^3^) corresponding to different volume fractions for each design type.

Number	Proportion of Steel Fiber Components with Different Lengths (kg/m^3^)					
	3 mm	6 mm	8 mm	10 mm	13 mm	16 mm
UHPC-CA-A1	0	0	0	0	78.50	0
UHPC-CA-A1.5	0	0	0	0	117.75	0
UHPC-CA-A2	0	0	0	0	157.00	0
UHPC-CA-A2.5	0	0	0	0	196.25	0
UHPC-CA-A3	0	0	0	0	235.50	0
UHPC-CA-B1	12.56	13.45	8.64	17.27	16.49	10.21
UHPC-CA-B1.5	18.84	20.02	12.95	25.91	24.73	15.31
UHPC-CA-B2	25.12	26.69	17.27	34.54	32.97	20.41
UHPC-CA-B2.5	31.40	33.36	21.59	43.12	41.21	25.51
UHPC-CA-B3	37.68	40.04	25.91	51.81	49.46	30.62
UHPC-CA-C1	18.47	25.40	13.85	9.24	6.93	4.62
UHPC-CA-C1.5	27.71	38.10	20.78	13.85	10.39	6.93
UHPC-CA-C2	36.94	50.79	27.71	18.47	13.85	9.24
UHPC-CA-C2.5	46.18	63.49	34.63	23.09	17.32	11.54
UHPC-CA-C3	55.41	76.19	41.56	27.71	20.78	13.85

**Table 6:** Complete mix proportions for all UHPC-CA specimens (kg/m^3^).

Number	Proportion of Each Raw Material (kg/m^3^)							Proportion of Steel Fiber Components with Different Lengths (kg/m^3^)					
	Cement	Silica Fume	Fly Ash	Fine Aggregate	Coarse Aggregate	Superplasticizer	Water	3 mm	6 mm	8 mm	10 mm	13 mm	16 mm
UHPC-CA-A1	843.5	234.3	93.7	1171.5	542.4	35.15	206.2	0	0	0	0	78.50	0
UHPC-CA-A1.5								0	0	0	0	117.75	0
UHPC-CA-A2								0	0	0	0	157.00	0
UHPC-CA-A2.5								0	0	0	0	196.25	0
UHPC-CA-A3								0	0	0	0	235.50	0
UHPC-CA-B1								12.56	13.45	8.64	17.27	16.49	10.21
UHPC-CA-B1.5								18.84	20.02	12.95	25.91	24.73	15.31
UHPC-CA-B2								25.12	26.69	17.27	34.54	32.97	20.41
UHPC-CA-B2.5								31.40	33.36	21.59	43.12	41.21	25.51
UHPC-CA-B3								37.68	40.04	25.91	51.81	49.46	30.62
UHPC-CA-C1								18.47	25.40	13.85	9.24	6.93	4.62
UHPC-CA-C1.5								27.71	38.10	20.78	13.85	10.39	6.93
UHPC-CA-C2								36.94	50.79	27.71	18.47	13.85	9.24
UHPC-CA-C2.5								46.18	63.49	34.63	23.09	17.32	11.54

**Table 7:** Test results of all UHPC-CA specimens (mean ± SD).

Number	Slump (mm)	Flow Spread (mm)	Compressive Strength (MPa)	Flexural Strength (MPa)	Splitting Tensile Strength (MPa)
UHPC-CA-A1	253 ± 6	388 ± 15	131.2 ± 3.5	20.7 ± 1.2	13.9 ± 0.8
UHPC-CA-A1.5	242 ± 8	367 ± 13	140.8 ± 3.8	23.6 ± 1.5	15.3 ± 1.0
UHPC-CA-A2	226 ± 5	335 ± 12	147.6 ± 4.3	26.9 ± 1.2	17.2 ± 1.1
UHPC-CA-A2.5	212 ± 5	312 ± 12	143.9 ± 4.1	24.6 ± 1.1	16.0 ± 1.0
UHPC-CA-A3	202 ± 6	294 ± 10	139.2 ± 3.9	22.1 ± 1.4	14.8 ± 0.9
UHPC-CA-B1	264 ± 8	401 ± 16	134.6 ± 3.7	24.0 ± 1.2	16.1 ± 0.7
UHPC-CA-B1.5	249 ± 7	372 ± 15	143.9 ± 4.6	25.9 ± 1.0	18.5 ± 1.2
UHPC-CA-B2	234 ± 6	358 ± 15	150.2 ± 4.1	30.2 ± 1.2	20.9 ± 0.9
UHPC-CA-B2.5	221 ± 8	334 ± 13	147.3 ± 3.5	27.2 ± 1.1	19.2 ± 0.7
UHPC-CA-B3	211 ± 6	309 ± 13	142.5 ± 3.6	24.9 ± 1.3	17.5 ± 1.3
UHPC-CA-C1	272 ± 9	413 ± 14	136.4 ± 3.8	25.4 ± 1.2	19.4 ± 1.4
UHPC-CA-C1.5	259 ± 7	392 ± 12	146.9 ± 4.3	28.7 ± 1.0	20.1 ± 1.2
UHPC-CA-C2	247 ± 6	370 ± 14	152.6 ± 5.2	31.8 ± 1.3	22.3 ± 0.8
UHPC-CA-C2.5	231 ± 5	355 ± 13	149.5 ± 3.9	29.6 ± 1.2	21.3 ± 1.2
UHPC-CA-C3	220 ± 5	330 ± 12	145.3 ± 3.5	27.3 ± 0.9	20.3 ± 0.9

## Data Availability

The original contributions presented in this study are included in the article. Further inquiries can be directed to the corresponding author.
